# Assessment of aflatoxigenic *Aspergillus* and other fungi in millet and sesame from Plateau State, Nigeria

**DOI:** 10.1080/21501203.2014.889769

**Published:** 2014-03-25

**Authors:** C.N. Ezekiel, I.E. Udom, J.C. Frisvad, M.C. Adetunji, J. Houbraken, S.O. Fapohunda, R.A. Samson, O.O. Atanda, M.C. Agi-Otto, O.A. Onashile

**Affiliations:** a Mycology/Mycotoxicology Research Unit, Department of Biosciences and Biotechnology, Babcock University, Ilishan Remo, Ogun State, Nigeria; b Department of Basic Sciences, Federal College of Animal Health and Production Technology, National Veterinary Research Institute, Vom, Jos, Nigeria; c Department of Systems Biology, Center for Microbial Biotechnology, Building 221, Technical University of Denmark DK-2800, Kgs. Lyngby, Denmark; d Department of Food Science and Technology, Federal University of Agriculture Abeokuta, Abeokuta, Ogun State, Nigeria; e CBS-KNAW Fungal Biodiversity Centre, Uppsalalaan 8, NL-3584 CT Utrecht, The Netherlands; f Department of Biological Sciences, McPherson University, Km 96, Lagos-Ibadan Expressway, Seriki-Sotayo, Near Ajebo Camp, Ogun State, Nigeria

**Keywords:** acha, aflatoxin, *Aspergillus*, food safety, fungi, fonio millet, sesame

## Abstract

Sixteen fonio millet and 17 sesame samples were analysed for incidence of moulds, especially aflatoxigenic *Aspergillus* species, in order to determine the safety of both crops to consumers, and to correlate aflatoxin levels in the crops with levels produced by toxigenic isolates on laboratory medium. Diverse moulds including *Alternaria, Aspergillus, Cercospora, Fusarium, Mucor, Penicillium, Rhizopus* and *Trichoderma* were isolated. *Aspergillus* was predominantly present in both crops (46–48%), and amongst the potentially aflatoxigenic *Aspergillus* species, *A. flavus* recorded the highest incidence (68% in fonio millet; 86% in sesame kernels). All *A. parvisclerotigenus* isolates produced B and G aflatoxins in culture while B aflatoxins were produced by only 39% and 20% of *A. flavus* strains isolated from the fonio millet and sesame kernels, respectively. Aflatoxin concentrations in fonio millet correlated inversely (*r* = −0.55; *p* = 0.02) with aflatoxin levels produced by toxigenic isolates on laboratory medium, but no correlation was observed in the case of the sesame samples. Both crops, especially sesame, may not be suitable substrates for aflatoxin biosynthesis. This is the first report on *A. parvisclerotigenus* in sesame.

## Introduction

Pre- and post-harvest contamination of crops by toxigenic fungi (e.g. species of *Aspergillus*) pose a threat to food safety due to liberation of toxic secondary metabolites (e.g. aflatoxins). Aflatoxins are the most important group of mycotoxins due to the severe health risks associated with their exposures in animals and humans (Bankole et al. 2006). The concern for food safety due to aflatoxin contamination by toxigenic *Aspergillus* is more pronounced in Sub-Saharan Africa, as compared to the temperate regions, where important food staples such as maize and groundnut are regarded as prone to aflatoxin contamination (Bankole and Adebanjo 2003; Bankole et al. 2006). Reasons for this include the hot and humid tropical climate which provides the toxigenic species with favourable conditions for growth. Consequently, researchers are investigating other cereal and oil-seed crops that may be less susceptible to aflatoxin contamination. Food crops such as fonio millet and sesame, which are not as widely consumed, have the potential to serve as safer food crops. Even though *Aspergillus flavus* has been reported to be the major post-harvest colonizer of both crops, it has been reported that these crops usually have very low or no aflatoxin levels present (Gbodi et al. 1986; Mbah and Akueshi 2000, 2009; Makun et al. 2007; Amadi and Adeniyi 2009; Asadi et al. 2011; Diedhiou et al. 2011; Ezekiel et al. 2012).

Fonio millet, *Digitaria exilis* (Kippist) Stapf, which is known as “acha” in Nigeria, is among the oldest of West African cereals and one of the four millets grown in the savannahs of Africa (Cruz 2004). However, fonio millet is regarded as a neglected cereal and constitutes less than 0.1% and 0.25% of staple foods and total grains produced in Nigeria, respectively (FAOSTAT 2012). More than 95% of northerners in Nigeria and neighbouring countries consume fonio millet as a staple in the form of porridges, and also as beverages like “kuunu.” This is possibly due to its high amino acid and mineral contents, and low glycaemic index, which makes it a good substitute for other cereal-based diets and a suitable food for diabetics (Nzelibe et al. 2000; Belton and Taylor 2002; O'Kennedy et al. 2006).

Sesame (Benniseed; *Sesame indicum* L.) is an oil seed grown in the savannah zones of Nigeria and in many other parts of the world (Alegbejo et al. 2003), whose oil is often preferred to the commonly available groundnut oil due to its better flavour-enhancing property, while the seeds are utilized as spices and livestock feed. Sesame is also a major cash crop exported from Nigeria to countries like Japan and Turkey (Chemonics 2002).

As a follow-up to a previous study in which 55 multi-microbial metabolites were detected in fonio millet and sesame samples (Ezekiel et al. 2012), this study was designed to provide reliable data on the mycobiota associated with both crops. Both foodstuffs were obtained from farmers’ stores and assessed for the presence of fungi, especially the aflatoxigenic *Aspergillus* species. In this study, the *in vivo* production levels of aflatoxin were compared to those produced *in vitro* by sampled *Aspergillus* isolates in order to determine the suitability of each crop to serve as a substrate for aflatoxin contamination.

## Materials and methods

### Samples

Sampling and treatments were done according to Ezekiel et al. (2012). Sixteen fonio millet and 17 sesame samples were collected from farmers’ stores in Plateau State, Nigeria between April and May 2011. Only samples processed and stored for less than 30 days after harvest were collected. Each bulk sample (1.8–2 kg) was quartered and about 120 g of representative sample was obtained by randomly selecting a quarter. Representative samples were ground in a Waring blender, stored at 4°C and analysed within 24 hours.

### Isolation of fungi from fonio millet and sesame kernels

Moulds occurring in the milled fonio millet and sesame samples were isolated by the dilution plating technique described by Samson et al. (1995). Briefly, 10 g of each sample was suspended in 90 ml of sterile distilled water and homogenized for 2 min. A 0.1 ml aliquot was spread-plated in triplicate on a set of four semi-selective mycological media: peptone pentachloronitrobenzene agar (PPA) (Nash and Snyder 1962); modified rose bengal agar (mRBA) (Cotty 1994); dichloran rose bengal chloramphenicol agar (DRBC) (Pitt and Hocking 2009); and one-quarter strength potato dextrose agar (PDA), 9.75 g/l PDB (Difco) and 20 g/l bacto agar] supplemented with 0.002% lactic acid. All plates of mRBA were incubated for 3 days at 31°C while DRBC, PDA and PPA plates were incubated at 25°C for 5–7 days. Colonies of *Aspergillus, Fusarium, Penicillium* and *Talaromyces* were counted on mRBA, PPA and DRBC plates, respectively, while all moulds including *Alternaria, Cercospora* and the Mucorales were counted on PDA plates. Mould load per sample was derived from plate counts and expressed as a logarithm of colony-forming units per gram of sample (Log_10_CFU/g).

### Fungal identification

Colonies of *Aspergillus* were transferred to 5/2 agar (5% V-8 juice and 2% agar, pH 5.2) and malt extract agar (MEA), while *Penicillium* and *Talaromyces* were transferred to water agar (2% agar in distilled water). Colonies belonging to *Fusarium, Alternaria, Cercospora* and the Mucorales were purified on full-strength PDA. For section *Flavi,* 20 isolates were transferred to 5/2 agar from triplicate mRBA plates belonging to each sample.

All isolates were subjected to morphological identification by assessing macroscopic and microscopic characters in line with appropriate keys. Isolates belonging to *Aspergillus* section *Flavi* were identified to species level (Cotty and Cardwell 1999; Klich 2002; Frisvad et al. 2005; Ehrlich et al. 2007), while all other fungi (including other *Aspergillus* isolates) were identified to the genus level (Samson et al. 1995; Frisvad and Samson 2004; Leslie and Summerell 2006; Pitt and Hocking 2009; Samson et al. 2010, 2011).

### Characterization of *Aspergillus* section Flavi

A combination of phenotypic character assessment and aflatoxin production was used for the identification of *Aspergillus* section *Flavi* isolates obtained from both crops. Assessment of aflatoxin production was performed on neutral red desiccated coconut agar (NRDCA) as described in Ezekiel et al. (2013b). Isolates were identified as *A. flavus* if they met the following conditions: smooth surface conidia (magnification ×400), production of large sclerotia (>400 μm diameter) or no sclerotia on 5/2 agar, and production of B aflatoxins or were atoxigenic on NRDCA. *Aspergillus tamarii* was identified as brown to yellow-brown colonies of atoxigenic isolates, which produced rough conidia (magnification ×400) on 5/2 agar. All identified isolates were stored at 4°C as 5/2 agar plugs in sterile water. Isolates with numerous small sclerotia (400 μm average diameter) on 5/2 agar, and that produced B and G aflatoxins on NRDCA, were tentatively identified as *A. parvisclerotigenus.*

A molecular confirmation of the phenotype-based (morphological and biochemical) identifications was performed using six representative isolates of *A. parvisclerotigenus*. The total genomic DNA of the isolates was extracted, and a part of the calmodulin and β-tubulin genes were subsequently amplified and sequenced according to the methods previously established by Houbraken et al. (2011, 2012). The obtained sequences were queried in the NCBI sequence database and internal databases of the CBS-KNAW Fungal Biodiversity Centre for confirmation of species identity. The *A. parvisclerotigenus* isolates were incorporated in the CBS culture collection under accession numbers CBS135404–CBS135406 and CBS135587–CBS135589. Sequences were deposited in the NCBI nucleotide sequence database under accession numbers KF543328–KF543339.

### Aflatoxigenicity assay of *Aspergillus* section *Flavi* isolates

Each identified isolate belonging to the section *Flavi* was tested for *in vitro* aflatoxin production on NRDCA as mentioned above. After incubating the NRDCA plates for 5 days at 31°C, aflatoxin production was determined qualitatively and quantitatively using the thin-layer chromatography method outlined in Atanda et al. (2011).

### Aflatoxin determination in fonio millet and sesame kernels

All milled samples of fonio millet and sesame were analysed for multi-microbial metabolites, including aflatoxins, using a simple liquid chromatography tandem mass spectrometric method as reported in a previous paper (Ezekiel et al. 2012). The aflatoxin concentration data obtained for both crops from the multi-microbial metabolite screening paper were considered in the present study.

### Data analysis

Data obtained from this study were analysed using SPSS® 15.0 (Windows version, SPSS, IL, USA). Mould loads in the samples were calculated as log_10_CFU/g. The means of values obtained for the distribution of *Aspergillus* section *Flavi* species, and proportion of toxigenic to atoxigenic (tox:atox) *A. flavus* strains, were tested for significance at 95% confidence level using one-way ANOVA. The Duncan's Multiple Range Test (DMRT) was used to separate the means. Correlation analysis was performed to determine the relationship between the following: (1) proportion of tox and atox isolates and aflatoxin concentration in both crops, and (2) concentrations of aflatoxin produced by toxigenic isolates *in vitro* and aflatoxin levels in both crops.

## Results and discussion

### Occurrence of fungi in fonio millet and sesame kernels

All samples of fonio millet (*n* = 16) and 15 out of 17 (88.7%) sesame samples showed the presence of fungal propagules (Table 1). Diverse moulds including species of *Alternaria, Aspergillus, Fusarium* and *Penicillium* were isolated from both crops at varying proportions (Table 1). In addition, *Cercospora* spp. was found only in sesame while species of Mucorales (*Mucor* and *Rhizopus*), *Talaromyces* and *Trichoderma* were isolated from both fonio millet and sesame, and was regarded as “Others” in Table 1. Fungal occurrence data from both crops showed that *Aspergillus* spp. dominated (46–48%) followed by *Fusarium* spp. (30–42%). *Penicillium* spp. (11.5%) followed in fonio millet while the occurrence of *Cercospora* spp. (5.0%) was much less than that of *Fusarium* spp. (41.6%) in sesame (Table 1). Overall, mould load was significantly higher in fonio millet (range = 2.30–4.88, mean = 4.12 ± 0.64 log_10_CFU/g) than in sesame (range = 2.48–3.98, mean = 2.97 ± 1.09 log_10_CFU/g). The diverse fungal species isolated from the fonio millet and sesame samples were previously reported in finger millet from Nigeria (Makun et al. 2007) and sesame from Sierra Leone and Sudan (Jonsyn 1988; Khamees and Schlosser 1990). Only *Aspergillus, Fusarium* and *Penicillium* species were previously reported to invade and colonize post-harvest sesame in Nigeria (Gbodi et al. 1986; Mbah and Akueshi 2000). This article is the first report on the occurrence of *Alternaria* and *Cercospora* spp. in sesame from farmers’ stores in Nigeria.

**Table 1.** Mould load and occurrence of fungal genera in fonio millet and sesame from Plateau State, Nigeria.

		Mould load (Log_10_CFU/g)		Percentage occurrence of fungal genera in fonio millet and sesame seeds					
Crop	Nc^1^ (%)	Range	Mean^2^ ± SD	*Alternaria*	*Aspergillus*	*Cercospora*	*Fusarium*	*Penicillium*	*Others*
Fonio millet	16/16 (100.0)	2.30–4.88	4.12 ± 0.64a	8.0	46.0	0.0	30.2	11.5	4.3
Sesame	15/17 (88.7)	2.48–3.98	2.97 ± 1.09b	0.7	48.1	5.0	41.6	1.5	3.1

Notes: ^1^Number of contaminated samples.

2 Means with different alphabets in a column are significantly different (*p* < 0.05).

### Incidence of species within *Aspergillus* section *Flavi*

The contamination level and occurrence of species within section *Flavi* are presented in Table 2. Sesame kernels (range = 2.0–3.1, mean = 2.53 ± 0.44 log_10_CFU/g) had a higher but statistically insignificant load of section Flavi species than fonio millet (range = 1.7–2.9, mean = 2.41 ± 0.44 log_10_CFU/g). For 8 out of 16 fonio millet samples, 160 isolates representing *Aspergillus* section *Flavi* were collected while 220 isolates were recovered from 11 out of the 17 sesame samples. The section Flavi isolates found in this study included three species: *Aspergillus flavus, A. tamarii* and *A. parvisclerotigenus.* Among these species, *A. flavus* (68–86% incidence) predominated in both crops followed by *A. tamarii* (14–18% incidence). *A. parvisclerotigenus* was isolated from only sesame (incidence = 13.6%). Previous studies have shown that section *Flavi* members are common colonizers of all types of millet and sesame during post-harvest storage (Jonsyn 1988; Khamees and Schlosser 1990; Mbah and Akueshi 2000, 2009; Makun et al. 2007; Amadi and Adeniyi 2009; Diedhiou et al. 2011); however, this is the first report of *A. parvisclerotigenus* in sesame. No incidence of *A. parasiticus* was found in either fonio millet or sesame, which supports previous reports for sesame, but fails to support the findings of Makun et al. (2007) who reported finding *A. parasiticus* in finger millet from Niger State, Nigeria.

**Table 2.** Incidence of *Aspergillus* section *Flavi* species in fonio millet and sesame seeds from Plateau State, Nigeria.

		Load^2^ (Log_10_CFU/g)			Occurrence^5^ (%) of species in crops		
Crop	Nc^1^ (%)	Range	Mean^3^ ± SD	N^4^	*A. flavus*	*A. parvisclerotigenus*	*A. tamarii*
Fonio millet	8/16 (50.0)	1.70–2.90	2.41 ± 0.44	160	138a (86.3)	0b (0.0)	22b (13.7)
Sesame	11/17 (64.7)	2.00–3.08	2.53 ± 0.44	220	150a (68.2)	30b (13.6)	40b (18.2)

Notes: ^1^ Number of contaminated samples.

2 Load of *Aspergillus* section *Flavi* in the samples.

3 No statistical difference (*p* > 0.05) was obtained for mean load of the section *Flavi* in both crops.

4 Number of *Aspergillus* section *Flavi* isolates obtained from the contaminated samples.

5 Mean occurrence of species with different alphabets in a row are significantly different (*p* < 0.05).

### In vivo *and* in vitro *aflatoxigenicity of* Aspergillus *isolates*

None of the *A. tamarii* isolates (*n* = 62) obtained from the fonio millet and sesame samples produced aflatoxins. Conversely, all 30 isolates of *A. parvisclerotigenus* obtained from sesame kernels were aflatoxigenic, producing both B and G aflatoxins. In total, 138 and 150 *A. flavus* isolates were obtained from fonio millet and sesame kernels, respectively. For fonio millet, 39.1% of the isolates, and 20.0% of isolates from sesame kernels, produced B aflatoxins on NRDCA (Table 3). In both crops, the incidence of atoxigenic *A. flavus* isolates was significantly higher than that of toxigenic isolates (Table 3). Similarly, when all *Aspergillus* section *Flavi* isolates (*A. flavus, A. parvisclerotigenus* and *A. tamarii*) are considered, 27.3% and 33.8% of the isolates from sesame and fonio millet kernels, respectively, were toxigenic strains (data not shown). This indicates a higher proportion of atox:tox strains for the entire section *Flavi* community in both crops. A similar trend was reported for sesame in Senegal (Diedhiou et al. 2011). It may be appropriate to suggest that susceptibility of both crops, especially sesame, to contamination by aflatoxigenic strains is low compared to other crops/foods (e.g. maize, nutmeg and mushrooms) from Nigeria that showed higher tox:atox proportions (Atehnkeng et al. 2008; Ezekiel et al. 2013a, 2013c).

**Table 3.** Proportion (%) of toxigenic to atoxigenic *A. flavus* in fonio millet and sesame seeds from Plateau State, Nigeria.

	Mean proportions by crop^1^	
	Fonio millet	Sesame
Atoxigenic	60.9a	80.0a
Toxigenic	39.1b	20.0b

Note: ^1^ Means with different alphabets in a column are significantly different (*p* < 0.05).

Table 4 shows the concentrations of aflatoxins produced by toxigenic isolates of *A. flavus* and *A. parvisclerotigenus* on NRDCA. Toxigenic *A. flavus* from fonio millet produced higher amounts (range = 233.2–692 μg/kg; mean = 433.9 μg/kg) of B aflatoxins than those from sesame (range = 75.8–326.1 μg/kg; mean = 190.1 μg/kg). However, the levels of B aflatoxins produced by *A. parvisclerotigenus* from sesame was very high (mean = 601.3 μg/kg); therefore, increasing significantly the total B aflatoxin levels produced by toxigenic isolates of *Aspergillus* section *Flavi* from sesame than those from millet. The level of G aflatoxins (mean = 863.9 μg/kg) was higher than that of B aflatoxins. Minisclerotial species within the section *Flavi* have been reported to produce copious amounts of aflatoxins, especially the G aflatoxins (Egel et al. 1994; Atehnkeng et al. 2008; Donner et al. 2009), which is supported by the findings of the present study.

**Table 4.** Levels of aflatoxins produced by aflatoxigenic species from fonio millet and sesame in Plateau State, Nigeria.

	Concentration (μg/kg) of aflatoxins from toxigenic species						
		*A. flavus*			*A. parvisclerotigenus*		
Crops		B	G	B	G	Total B	Total G
Fonio millet	Range	233.2–692.0	–	–	–	233.2–692.0	–
	Mean^1^	433.9a	–	–	–	433.9b	–
Sesame	Range	75.8–326.1	–	215.6–1011.2	363.2–1980.7	75.8–1011.2	363.2–1980.7
	Mean^1^	190.1b	–	601.3	863.9	597.1a	863.9

Note: ^1^ Means with different alphabets in a column are significantly different (*p* < 0.05).

Only 13 out of the 16 (81.3%) fonio millet samples had aflatoxin contamination and the concentrations ranged 0.08–1.4 μg/kg, while none of the sesame samples had aflatoxin contamination. Toxigenic strains that are able to form aflatoxins *in vitro* on NRDCA were present for both crops. However, aflatoxins were absent or present in small amounts (Ezekiel et al. 2012) and suggest that these crops are not suitable substrates for aflatoxin biosynthesis. Several factors such as environmental conditions (e.g. moisture and temperature), agronomic practices for crop production and post-harvest treatment of the grains (e.g. processing, drying and storage practices) are known to influence fungal development and secondary metabolite formation in agricultural products. In this study, grain size, presence of inhibitory compounds towards aflatoxin production and duration of storage may have played significant roles in the low aflatoxin levels found in both crops. It may be best to associate the finding under discussion with mostly grain size, since it is observed that fonio millet is slightly bigger in size than sesame and had higher fungal contamination/load, incidence of toxigenic strains and aflatoxin content. A similar suggestion was given by Makun et al. (2013) who also found higher levels of ochratoxin A (OTA) in grains with larger sizes than acha and sesame. The presence of sesamin, a natural potent antifungal and anti-OTA chemical, in sesame may also have contributed to the absence of aflatoxins in this study as compared to the high levels of B and G aflatoxins produced on NRDCA. Mbah and Akueshi (2009) reported that only 20% of the 20 *A. flavus*-inoculated *Sesamum indicum* seeds had aflatoxin B_1_ content after 20 days of incubation; all other 40 inoculated samples of the same sesame species that were incubated for 10 and 15 days, as well as all 60 inoculated samples of *S. rediatum* incubated for 10, 15 and 20 days, did not show the presence of any aflatoxin. This further supports our finding that sesame is not a convenient substrate for aflatoxin production as previously suggested by Diedhiou et al. (2011). An additional militating factor may have been the short duration (less than 30 days) of seed storage.

### Correlation studies

No correlation was observed among the incidence of aflatoxigenic isolates from sesame, the aflatoxin concentration produced by the toxigenic isolates and the levels of aflatoxins in sesame, since none of the aflatoxins was detected in sesame grains. However, a positive (*r* = 0.70) significant (*p* = 0.02) correlation was observed between incidence of aflatoxigenic isolates from fonio millet and aflatoxin concentration *in vivo,* and significant (*p* = 0.03) inverse correlation (*r* = −0.41) between incidence of atoxigenic isolates and aflatoxins in the fonio millet. A significant (*p* = 0.02) inverse correlation (*r* = −0.55) was also observed between the levels of aflatoxins produced by toxigenic isolates on NRDCA and aflatoxins in fonio millet. The patterns obtained from the correlational study further confirms that though toxigenic isolates may be present among the community of *Aspergillus* colonizing sesame and fonio millet, their toxin potentials on these crops are reduced as compared to suitable media (e.g. NRDCA). The implication of this to consumer safety may be that both crops, and especially sesame, are most likely to be safe grains for consumption in Nigeria. With respect to aflatoxin contamination, no study has reported aflatoxin levels in both crops beyond the maximum tolerable limit of 20 μg/kg recommended for foods in Nigeria (Gbodi et al. 1986; Mbah and Akueshi 2009; Ezekiel et al. 2012). Further studies may consider evaluating the correlation between *in vitro* toxigenicity of other fungal species and relevant mycotoxins in both crops, though very low levels of other mycotoxins were found (e.g. deoxynivalenol, fumonisins and zearalenone) in both crops (Ezekiel et al. 2012).

## Conclusion

This study has shown that fonio millet and sesame, grown and stored by farmers in Plateau State, Nigeria, are susceptible to invasion and colonization by diverse moulds including aflatoxigenic isolates of *Aspergillus.* However, it is possible that aflatoxin contamination of both crops may not be a major issue, possibly due to grain size and natural inhibitory compounds in the grains. The major concern may be that of toxigenic isolate carry-over and distribution from these reservoir crops to other co-stored food materials in farmers’ store. Therefore, efforts in good storage and processing practices should be intensified as preventive measures against toxigenic strain dispersal.
